# The ecological determinants of baboon troop movements at local and continental scales

**DOI:** 10.1186/s40462-015-0040-y

**Published:** 2015-07-01

**Authors:** Caspian Johnson, Alex K Piel, Dan Forman, Fiona A Stewart, Andrew J King

**Affiliations:** Department of Biosciences, College of Science, Swansea University, Swansea, UK; Division of Biological Anthropology, Department of Archaeology and Anthropology, University of Cambridge, Cambridge, UK

**Keywords:** Day path length, Baboon, *Papio cynocephalus*, Season, Space-use, Ranging, Modelling, Speed, Turning angle, Human-modified habitat, Movement characteristics, Comparative analysis

## Abstract

**Background:**

How an animal moves through its environment directly impacts its survival, reproduction, and thus biological fitness. A basic measure describing how an individual (or group) travels through its environment is Day Path Length (DPL), i.e., the distance travelled in a 24-hour period. Here, we investigate the ecological determinants of baboon (*Papio* spp.) troop DPL and movements at local and continental scales.

**Results:**

At the continental scale we explore the ecological determinants of annual mean DPL for 47 baboon troops across 23 different populations, updating a classic study by Dunbar (Behav Ecol Sociobiol 31: 35-49, 1992). We find that variation in baboon DPLs is predicted by ecological dissimilarity across the genus range. Troops that experience higher average monthly rainfall and anthropogenic influences have significantly shorter DPL, whilst troops that live in areas with higher average annual temperatures have significantly longer DPL. We then explore DPLs and movement characteristics (the speed and distribution of turning angles) for yellow baboons (*Papio cynocephalus*) at a local scale, in the Issa Valley of western Tanzania. We show that our continental-scale model is a good predictor of DPL in Issa baboons, and that troops move significantly slower, and over shorter distances, on warmer days. We do not find any effect of season or the abundance of fruit resources on the movement characteristics or DPL of Issa baboons, but find that baboons moved less during periods of high fruit availability.

**Conclusion:**

Overall, this study emphasises the ability of baboons to adapt their ranging behaviour to a range of ecological conditions and highlights how investigations of movement patterns at different spatial scales can provide a more thorough understanding of the ecological determinants of movement.

**Electronic supplementary material:**

The online version of this article (doi:10.1186/s40462-015-0040-y) contains supplementary material, which is available to authorized users.

## Background

A simple, but revealing measure of an animal’s space use is the distance it moves within a 24-hour period. This distance is described as the Day Path Length (DPL). The simple parameters required to quantify DPL make it easily transferable and applicable to terrestrial and/or arboreal animals [1,2], thus affording comparative investigations of DPL across species. For example, DPLs provide the basis of analyses of mammalian day range [3], and some of the most comprehensive studies of what determines how far animals travel have been undertaken on primates [4]. Like most mammals, primate ranging behaviours are primarily influenced by the distribution and abundance of essential resources [4-6], specifically food [7], but a suite of other factors are also important.

In general, primates tend towards an energy maximising strategy [8] whereby, in response to low food availability, they increase their DPLs in search of higher quality food items [9-13]. Since plant biomass and net plant productivity can be reliably inferred from rainfall data [14,15], especially in seasonal habitats [16], rainfall can be used as an indirect measure of food resources and predicts primate DPLs [7,17]. Similarly, recent studies have demonstrated that remotely sensed data, particularly the normalized difference vegetation index (NDVI), provides an adequate measure of photosynthetic activity and, therefore, vegetation structure [18], which can hence be used to further understand primate movement ecology [19]. Increasing primate group sizes also results in longer DPLs [20] since larger groups experience greater intragroup feeding competition [21] and exhaust food patches quicker, forcing more frequent travel between patches [21-23]. Note, however, that primates with a more leaf-based and herbaceous diet lessens the effect of group-size on DPLs because the spatial-temporal distribution of leaves is more homogenous (e.g. *Brachyteles arachnoides hypoxanthus*, [24]; *Colobus badius tephrosceles*, [5]; *Gorilla* spp., [25,26]).

Baboons (*Papio* spp.) range throughout sub-Saharan Africa, across a multitude of habitat types making them the most widespread African primate genus [27] and perhaps coincidentally, are one of the best studied primates, particularly with respect to DPL. Numerous studies have shown that baboon DPLs respond to extrinsic changes in biotic and abiotic factors, attributed to the highly seasonal environments in which they live [9-13], and also to intrinsic social factors [11,28]. Accordingly, baboon troop DPLs across their range can be reliably predicted by group size and rainfall, as shown by a classic study by Dunbar in 1992 [29].

Since Dunbar’s original study [29] there have been further studies of the climatic determinants of foraging and ranging behaviour in baboons (e.g. [30-32]), and new data on baboon DPL and ecology now exist. We therefore revisit the question of what determines baboon troop DPLs at a continental scale with the addition of 29 data points (DPLs) taken from recent literature, whilst considering additional ecological variables. We adopt a mixed modelling/model selection approach instead of the stepwise linear regression approach used originally [29], and also consider the potential impact of anthropogenic influence, primate species number, and NDVI. We consider anthropogenic influence because where baboons rely on predictable and high-quality food sources (e.g. crops or food/waste) that occur in human modified habitats (e.g. [20-22]), DPLs are found to be reduced and not predicted well by models that include rainfall and group size as predictors [33]. We consider primate species number on the basis that a high number of primate species may result in increased levels of inter-specific competition, which is known to drive longer DPLs, especially in frugivorous primates (e.g. [20,21,30,34]). Additionally, as a more recent technological development, not available to Dunbar in his 1992 study, we also consider NDVI data as it provides a good proxy for photosynthetic activity and vegetation structure for study sites [19,35].

Our understanding of the ecological determinants of baboon day path lengths at a finer (local) scale comes primarily from arid savannah habitats [9,36-40], even when considering more recent studies on the topic [13,22,33,41-46]. To provide a fuller analysis of the ecological determinants of movement at a local scale, and to complement our continental scale analyse (see above), we investigated the daily movements of two troops of yellow baboons, *Papio cynocephalus*, inhabiting the primate-rich, seasonal, and predominantly woodland habitat of the Issa Valley in Ugalla, western Tanzania. This represents the first study of baboons in this region. We begin by exploring how well our inter-population model predicts DPLs for the Issa baboons, and then go on to consider what local ecological factors predict variation in DPLs and movement characteristics.

Variation in food resources are predicted to have a large effect on baboon space use. The proportion of fruit-based versus leaf-based forage in the diet, in particular, can have a large effect upon day ranges, with DPL increasing with the quantity of fruit in the diet [4]. Since fruit tends to grow ephemerally in small, finite patches, which are distributed heterogeneously, it is quickly exhaustible [23,47] and necessitates longer DPLs. Reliance on high-quality fruit can also drastically alter movement characteristics to maximise efficiency [21] and primates foraging on fruit show faster [48], straighter, and more goal-directed movement characteristics [49-51]. In contrast, leaf-based and herbaceous foods have a more homogeneous distribution in space and time [26] affording shorter DPLs and slower, more tortuous movement [52,53]. Regardless of food type, food abundance is dependent upon local, temporal variation in climate [16,54], and when food is scarce, individuals typically increase their DPLs in search of these food items (e.g. *Papio hamadryas*, [43]; *Papio anubis*, [12]; *Eulemur rubriventer* and *Eulemur fulvus rufus*, [55]; *Gorilla gorilla*, [56]; *Rhinopithecus* sp., [57]; *Colobus satanas*, [58]; *Cercocebus galeritus*, [59]). We therefore expected the baboons at Issa to demonstrate slower, less direct travel, and an increased DPL in times of reduced fruit availability [9-13].

Other climatic variables can also directly influence primate, and specifically baboon, ranging behaviour. If temperatures are too low, or too high, for example, primates reduce time spent travelling in order to conserve energy (e.g. *Rhinopithecus bieti*, [60]; *Papio ursinus*, [61]). Thus, ambient temperature can be an important climatic constraint on primate ranging behaviour, and we therefore tested the prediction that the baboons DPLs will be constrained by maximum daily temperatures in the warm Tanzanian climate, resulting in slower movement [32] and reduced DPL [61]. Finally, given that Issa’s baboons experience distinct wet and dry seasons, we also tested for any effect of season that might have additional and independent effects upon DPLs and movement characteristics because, for example, the availability of water sources change [9].

## Methods

### Continental scale

#### Data collection

For our continental scale analysis we used data provided in Dunbar’s (1992) study [29] and updated this with DPLs of 29 more recent studies from the literature (see Additional file 1). If data were available for more than one group at a study site, we use each troop’s DPL, and we collected information on the rate at which troop locations were taken throughout the day, i.e. sampling frequency, and whether annual mean DPL was calculated from >12 months study, <12 months, or if this was unknown, i.e. sample size. This enabled us to test for/control for any potential effect of differences in how annual mean DPL were estimated across studies in our analyses. We also collected information on troop size, anthropogenic influence (whether or not the diet of the troop was supplemented by human derived foods [yes/no]) and the number of primate species occurring at each study site. Nocturnal primates were included in the primate species count so as to account for any indirect competition that may result from their spatial overlap with the baboons. These ecological data for each study site are summarised in Table 1, and troop specific data on group sizes and DPLs are summarised in Additional file 1.

**Table 1:** **Ecological data for the 23 baboon populations used in the DPL continental comparison model**

**Species & study site**	**Latitude**	**Longitude**	**Altitude**	**#Study troops**	**#Primate Spp.**	**Anthropogenic influence?** ^**1**^	**References** ^**2**^
*Papio anubis*							
Bole, Ethiopia	9.42	38.00	1700	1	4	No	[80]
Budongo, Uganda	1.93	31.67	700	1	7	No	[113]
Chololo, Kenya	0.40	36.95	1660	1	2	No	[79]
Gashaka Gumti, Nigeria	7.51	11.61	320	2	9	Yes (1/2)	[114]
Gilgil, Kenya	−0.49	36.32	1770	1	1	No	[12]
Ishasha, Uganda	−0.62	29.66	950	1	4	No	[115]
Metahara, Ethiopia	8.91	39.93	950	1	2	No	[116]
Mulu, Ethiopia	9.30	40.83	1275	1	2	No	(Dunbar, *unpublished*)
*Papio cynocephalus*							
Amboseli, Kenya	−2.64	37.25	1130	6	3	Yes (1/6)	[17,33,117]
Mikumi, Tanzania	−7.09	37.42	550	1	5	No	[77]
Tana, Kenya	−1.93	40.14	30	1	6	No	[118]
Issa, Tanzania*	−5.51	30.56	1600	2	6	No	This study
*Papio hamadryas*							
Awash, Ethiopia	8.84	40.01	950	5	2	No	[43,45,119]
Erer-Gota, Ethiopia	9.56	41.38	1200	1	1	No	[120]
*Papio papio*							
Mt. Assirik, Senegal	12.87	−12.80	150	2	6	No	[38]
*Papio ursinus*							
Blouberg, SA	−23.03	29.06	900	1	3	No	(Noser, *unpublished*)
Cape Point, SA	−34.27	18.43	50	10	1	Yes (7/10)	[13,39,121]
Drakensberg, SA	−29.47	29.26	2250	2	1	No	[41,122]
Honnet, SA	−22.63	30.18	310	2	2	Yes (1/2)	[61]
Mkuzi, SA	−27.60	32.05	125	1	2	No	[42] from [123]
Suikersbosrand, SA	−26.50	28.22	1600	1	2	No	[124]
Tsaobis, Namibia	−22.55	15.73	1000	1	1	No	[44]
DeHoop, SA	−34.43	20.57	10	2	1	No	(Hill, *unpublished*)
Mt. Zebra, SA	−32.20	25.39	1500	1	1	No	[125] from [123]

^1^Indicates whether baboons studied experience anthropogenic influences, and if so, how many troops. ^2^Unpublished data are acquired from authors listed.

In keeping with previous comparative studies (e.g. [29-32,62]), we investigated the effect of the following climate variables on mean annual DPL: mean annual temperature (Tann), mean annual rainfall (Pann), variation (standard deviation) in monthly temperature (TmoSD), variation (standard deviation) in monthly rainfall (PmoSD), the number of months per year with less than 100 mm of rainfall (P < 100), and the primary productivity index (PPI: the number of months in the year where rainfall was more than twice the average annual temperature). PPI is a useful measure of productivity during the growing season in tropical habitats and is therefore a useful index of seasonality [62,63]. These climate data were taken from the original studies and/or Dunbar's (1992) study [29]; where this information was not available, we followed the methods provided in Bettridge et al. [31] and used data from the Willmott & Matsuura [64] meteorological database. This database provides a global dataset of annual and monthly temperatures and rainfall in grids of 0.5° latitude by longitude, which are derived from a combination of Legate and Willmott’s [65,66] weather station records and the Global Historical Climatology Network (version 2). We calculated average values across all data points in the Willmott & Matsuura dataset that fell within 0.5° latitude and longitude to the relevant site. All temperatures are provided in °C, and rainfall in mm. We also collected remotely sensed information on NDVI, since it is a well-established measure of photosynthetic activity and vegetation structure [18] with proven applications in understanding species’ ecology [19,35]. NDVI data was retrieved for an area of 10.25 km^2^ for each study site from the Oak Ridge National Laboratory Distributed Active Archive Centre (http://daac.ornl.gov/MODIS/) and a 14-year average for each site was calculated from the available MOD 13Q1 data set (2000–2014). All climate data for each specific baboon study site are summarised in Table 2.

**Table 2:** **Climate and environmental data for 23 baboon study populations**

**Species & study site**	**Pann**	**PmoSD**	**Tann**	**TmoSD**	**PPI**	**P < 100**	**NDVI**
*Papio anubis*							
Bole, Ethiopia	1105	85.75	19.50	1.30	8.0	8.0	0.47
Budongo, Uganda	1679	68.18	22.10	0.75	10.0	4.5	0.84
Chololo, Kenya	549	40.31	22.90	1.03	5.0	9.5	0.29
Gashaka Gumti, Nigeria	1800	109.90	26.60	1.00	8.0	5.0	0.38
Gilgil, Kenya	595	20.95	18.10	0.69	5.0	11.0	0.46
Ishasha, Uganda	1292	37.87	22.00	0.93	10.0	6.0	0.68
Metahara, Ethiopia	639	58.99	24.50	1.56	6.0	9.0	0.26
Mulu, Ethiopia	1105	64.00	15.90	1.61	8.0	7.0	0.42
*Papio cynocephalus*							
Amboseli, Kenya	336	23.44	22.86	1.43	3.0	11.0	0.26
Mikumi, Tanzania	832	63.27	25.21	2.72	6.0	6.0	0.6
Tana, Kenya	803	49.57	28.00	1.12	5.0	9.0	0.72
Issa, Tanzania*	1200	79.69	20.00	0.32	7.0	5.0	0.6
*Papio hamadryas*							
Awash, Ethiopia	639	49.28	24.62	1.68	6.0	8.8	0.28
Erer-Gota, Ethiopia	665	59.12	24.20	1.61	5.0	9.0	0.32
*Papio papio*							
Mt. Assirik, Senegal	953.9	97.90	30.50	2.45	5.0	7.7	0.47
*Papio ursinus*							
Blouberg, SA	343	35.42	20.75	3.67	7.0	12.0	0.50
Cape Point, SA	743	36.86	17.90	3.47	6.9	10.0	0.42
Drakensberg, SA	1197	82.57	14.60	4.18	8.3	6.0	0.45
Honnet, SA	307	45.01	21.33	3.58	3.0	10.3	0.29
Mkuzi, SA	630	37.77	22.40	2.92	6.0	9.8	0.68
Suikersbosrand, SA	700	44.42	15.95	4.50	7.0	9.0	0.56
Tsaobis, Namibia	122	16.45	13.80	2.33	3.0	12.0	0.12
DeHoop, SA	428	9.23	16.50	3.07	7.0	12.0	0.58
Mt. Zebra, SA	343	16.11	15.00	4.57	6.0	12.0	0.32

*Pann* average annual rainfall, *PmoSD* standard deviation for average monthly rainfall (mm), *Tann* average annual temperature (°C), *TmoSD* standard deviation for average monthly temperature (°C), *PPI* primary productivity index (number of months in the year in which rainfall was twice the average annual temperature), *P < 100* number of months with less than 100 mm rainfall, *NDVI* normalised difference vegetation index retrieved from remote sensing data. *Current study; not included in continental analysis.

#### Statistical analyses

We fitted annual mean DPL as the response variable in a linear mixed model (LMM) in R (lme4 package [67], R version 3.1.0) to determine which of the aforementioned ecological and climatic variables best explained variation in mean baboon troop DPLs. We fitted ‘population’ as a random effect to control for the potential non-independence of data from multiple troops within the same population. Co-linearity between all effects was checked using Spearman’s rank correlation tests, with a cut-off criterion of *r*_*s*_ = 0.60 [68] for including effects in the same model. We then fitted a series of models entering combinations of ecological and climate variables as continuous fixed and/or categorical fixed effects. Additional file 2 provides the top ten candidate models used to predict variation in annual mean DPL at a continental scale. To choose among models, we adopted a minimum adequate model selection procedure that considered all biologically meaningful combinations of the fixed effects described. Candidate models with the lowest Akaike information criterion (AIC) value [69] were consequently selected. Where models had AIC scores within two points of each other, both models were considered to be plausible alternatives and the model that was the most parsimonious (i.e. the model with the fewest fixed effects) was selected preferentially [70]. The significance of individual terms were then calculated from the selected model and terms not included in the selected model were put back into the model to obtain level of non-significance (lmerTest package, R: [71]).

### Local scale

#### Study site

Local scale data was collected in the Issa valley of western Tanzania (05° 23 S 30° 35 E), 81 km East of Lake Tanganyika. The Ugalla region extends over 3352 km^2^ and is comprised of steep, broad valleys and flat hilltop plateaus that range in altitude from 900 -1800 m. The habitat of the study area is described as being a diverse mixture of vegetation types including swamp, dry grassland, wooded grassland, woodland, gallery forest, thicket forest, and hill forest [72].

#### Movement data

Movement data were collected by CJ and field assistants from January to August 2012 in accordance with the regulations of the Tanzanian Wildlife Research Institute. In total 81 days were spent tracking two troops of yellow baboons over the study period. These were Matawi Troop (MT, N = 31 group members) and Camp Troop (CT, N = 22 group members). The baboons were successfully located on 61 of these tracking days. Once found, the troop was followed until they reached a sleeping site, typically around 19:00. Observers would then return the following morning to the same place at 07:00 (before baboons left the sleeping site). This was repeated until they were lost, or a full three days of follows were completed. In total this yielded a total observation time of 546 hrs (CT: 349 hrs, MT: 197 hrs). On all occasions the troops were followed, troop movement was recorded at 5-minute intervals, at a distance of 20-50 m behind the troop, using hand-held Garmin 520Hcx Global Positioning Systems (GPS). These GPS data were used to record the distance troops travelled from sunrise (07:00 ± 15 mins) to sunset (19:00 ± 30 mins).

To calculate DPLs, distances between consecutive GPS points were calculated using the Great-Circle Equation [73]. DPL’s were only calculated from full-day follows, or where the baboon locations were unknown for a period of less than 60 minutes representing a mean of 4.8 full day follows per month (CT: 3.1 days per month, MT: 1.7 days per month). Movement characteristics, as described by speed and turning angle distributions can provide information on orientation and searching behaviour [74]. Speed (m/min) and turning angle (θ) were calculated for successive GPS locations using the adehabitatLT package, R [75].

#### Temperature and season

**Figure 1:**
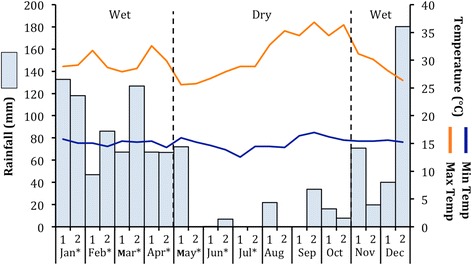
Issa climate. Minimum and maximum average bi-monthly temperatures and rainfall at Issa during 2012. The study period is depicted by months with an *. Months belonging to the dry season are those with <100 mm of rainfall and are highlighted with dashed lines.

An Onset H8 Pro series Hobo temperature logger was deployed in woodland plateau vegetation. This device recorded ambient temperature every 30 mins and provided minimum, maximum, and mean temperature readings daily (range: 12.5 – 38.7°C; mean ± Standard Deviation: 20.5°C ± SD 3.8°C: Figure 1). Rainfall was recorded using an Onset HOBO data logging rain gauge RG3-M deployed in the woodland plateau near camp. From January to July 2012, rainfall averaged 111 ± SD 93 mm/month, range: 0–248 mm (Figure 1). There were two distinct seasons at Issa, a rainy season (November-April) and a dry season (May-September), with dry months being defined as having <100 m of rainfall [72].

#### Food availability

**Figure 2:**
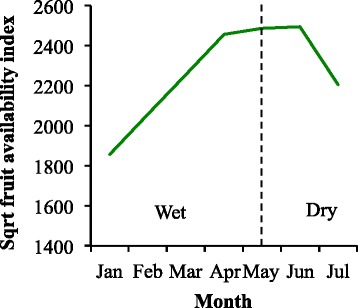
Fruit abundance. Fruit abundance at Issa for duration of study period. The dashed line represents the division between seasons.

Whilst baboons rely on a variety of food sources [40], fruit comprises a large portion of their diet [12,33,37-40,76-80] and is selected for when available [9]. We therefore utilised a pre-established phenology transect, that intersected the miombo woodland habitat, that was 1.7 km in length and 10 m in width and was fully contained within the home range of CT. Only woody plants known to produce fruits or seed pods that were consumed by the baboons and that were ≥ 2 m in height with a diameter at breast height ≥ 5 cm were monitored. This resulted in a total of 288 shrubs, lianas and trees from 17 species. The transect was walked every month for the duration of the study period, and the presence/absence of fruit or seed pods for each plant was noted [81]. Fruit abundance (we use this as a proxy for fruits and seed pods combined) was then estimated with a commonly used measure, the monthly fruit abundance index (FAI_*m*_) [82-85]:

$$ FA{I}_m={\displaystyle \sum_{k=1}^n}{D}_k{B}_k{P}_{km} $$

where D_*k*_ is the density of species *k* per km^2^, B_*k*_ is the mean DBH of species *k*, and P_*km*_ is the percentage of trees of species *k* in a fruiting condition in a month *m* (Figure 2).

#### Statistical analyses

To test for differences in DPLs of the two Issa troops, a Mann Whitney *U*-test was used. To investigate what factors predicted variation DPL we used a linear model (LM) (lme4 package, R: [67]), with normal error structure. We fitted a series of fixed effects in accordance with our predictions. Our two continuous effects were maximum temperature (°C) and FAI, and we fitted season (wet, dry), and troop ID as categorical effects. We used maximum temperature as a reflection of the hottest part of the day, which is most likely to constrain baboon DPL.

To test what factors predicted variation in speed and/or distribution of turning angles we implemented generalised additive models (GAM) (mgcv package, R: [67]). We only analysed speed and turning angle data where baboons were not stationary (i.e. speed > 1 m/min), and randomly sub-sampled n = 10 data points from each observation day to remove any temporal auto-correlation in our data. We then fitted maximum temperature, FAI and season (wet, dry) as fixed effects, whilst controlling for any effect of day (of study period) and troop (CT, MT). We used a GAM here rather than a standard linear model because GAMs are more capable of recognising nonlinear temporal variation [86]. The smoothed effect of time (day of study period) was based on penalized regression splines, to take into consideration the cyclic pattern of patterns of space-use.

For both our LMM (DPL analyses) and GAMs (speed, turning angle analyses) minimum adequate model selection was based on a procedure that considered all biologically meaningful combinations of fixed effects. The best model was subsequently selected by the lowest AIC value [69], but models within two AIC points were considered to be plausible alternatives and the model that was the most parsimonious (i.e. the model with the fewest fixed effects) was selected preferentially [70]. The significance of the individual terms was then calculated from the selected model and all dropped terms were put back into the model to obtain the level of non-significance (lmerTest package, R [71]).

## Results and Discussion

### Continental scale

**Figure 3:**
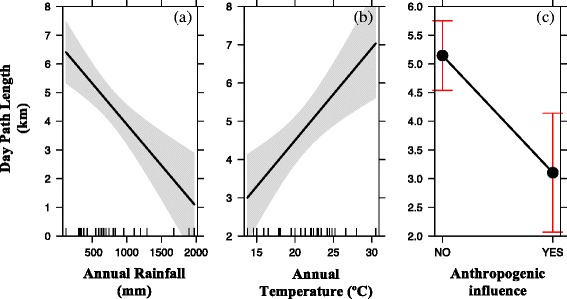
Predictors of DPL for N = 47 baboon troops across Africa. Significant effects of **(a)** average monthly rainfall (effect[SE] = 0.04[0.01]; df =1; p = <0.0003); **(b)** average annual temperature (effect[SE] = −0.23[0.06]; df =1; p = 0.001); **(c)** Anthropogenic influence (effect[SE] = −2.01[0.42]; df =1; p = 0.0001). Effects shown are predictions from our LMM (see Table 3) and upper and lower 95% confidence limits are indicated by shaded areas for **(a)** and **(b)** and whiskers for **(c)**.

Our analysis of the effects of ecological and biological variables on DPLs at a continental scale indicates that mean DPLs for 47 baboon troops across 23 different populations were best explained by a model that considered the independent effects of mean monthly rainfall, mean annual temperature, and anthropogenic influence (Table 3; Figure 3 and see Additional file 2 for best candidate models). All other fixed effects tested did not significantly predict variation in annual mean DPL (Table 3). We discuss each of the main effects in turn.

**Table 3:** **Estimate, standard error, test statistic and P-value for compatible predictors of annual mean DPL for baboon troops at a continental scale**

**Model term**	**Estimate**	**Standard error**	***t-*** **value**	***df***	***p***
Temperature (mean annual)	**0.24**	**0.07**	**3.61**	**1**	**0.002**
Rainfall (mean annual)	**−0.003**	**0.0007**	**−4.14**	**1**	**0.0005**
Anthropogenic influence^1^	**−2.04**	**0.46**	**−4.39**	**1**	**0.0001**
Sample size (months)^2^	0.34	0.79	0.44	2	0.08
Temperature (monthly SD)^3^	0.41	0.27	1.51	1	0.14
Troop size	0.005	0.005	0.92	1	0.36
Altitude	−0.0006	0.0005	−1.25	1	0.22
Sample frequency (GPS)^4^	−0.03	0.02	−1.65	1	0.14
NDVI	−0.84	1.89	−0.44	1	0.66

The best fitting model included those terms shown in bold text; for AIC values of the best candidate models tested see Additional file 2.

^1^Categorical effect (yes, no); reference category was no anthropogenic influence. ^2^Categorical effect representing whether the mean DPL was calculated from >12 months study, <12 months, unknown. ^3^Standard deviation in rainfall across months. ^4^The frequency of GPS fixes taken per hour to calculate DPL.

With higher mean monthly rainfall we found shorter baboon DPLs. As higher levels of precipitation typically result in more productive habitats and therefore more food [15,16], troops should encounter food more frequently and thus travel shorter distances at sites that experience high rainfall [9]. A more direct measure of vegetation (NDVI) did not, however, predict annual mean DPL. One possible reason for this might be because of baboons reliance on surface water, that they require on a daily basis [17], and whilst NDVI may represent “better” quality habitat, it does not necessarily reflect water availability, which might act as a constraint on baboon movement. We also found that baboons in hotter habitats travel further than those in cooler habitats. If the relationship between temperature and DPL in this case were causal, we would expect baboons to travel less far in hotter habitats, due to enforced rest as a result of thermal loading [87]. Instead, it is likely that higher ambient temperatures reflect more arid and therefore less productive environments with less surface water [88]. We therefore interpret the positive effect of hotter environments on annual mean DPL to be a consequence of variance in productivity and surface water across sites. Given the significance of annual temperature and monthly rainfall at this scale, it would be instructive to gather information on the availability of drinking sites/surface water in future work to quantify directly the importance of this resource in determining baboon DPL. We also found that DPLs were shorter where troops experienced anthropogenic influence. Anthropogenic influence was not considered by Dunbar [29] in his original model, but has since been highlighted as an important factor mediating DPLs [22,33]. This is because baboons in human-modified habitats typically have access to high quality and predictable food resources meaning baboons are able to sate their nutritional requirements within a smaller daily ranging distance, e.g. by crop-raiding and/or scavenging human foods [22,89-94].

Contrary to Dunbar [29] and our own expectations, we did not find that group size predicts variation in annual mean DPL. The negative effect of increasing group size on ranging behaviour has been well documented across the primate order [4,95] and within the baboon genus [11,28,29]. The lack of any group size effect here might be explained by the importance of the key ecological variables retained in our final model; these appear to be far more important, perhaps reflecting the changing environments and associated increase in exposure to human-modified habitats that baboons are experiencing. The effect of human-modified habitat use has also been reported to negate the effect of group size at a local scale. In the Cape Peninsula, South Africa, Hoffman & O’Riain [22] found that the largest group in the population (N = 115) had a DPL that did not differ significantly from the two smallest troops (both troops N = 16), which was explained by their near 100% use of human-modified habitat.

### Local scale

**Figure 4:**
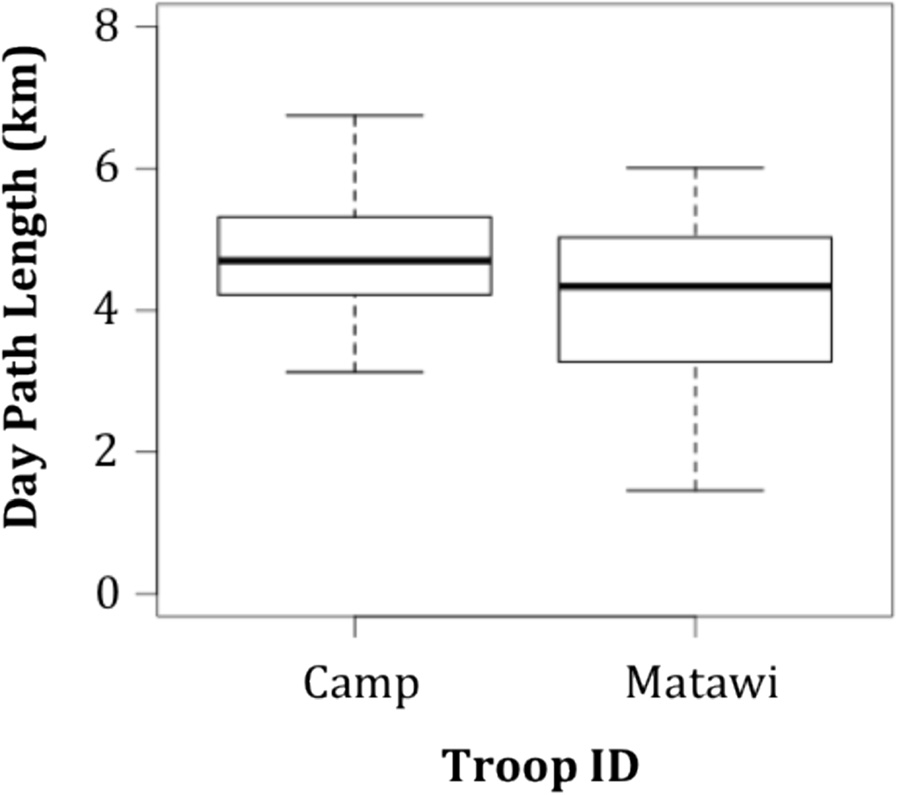
DPL of Issa baboon troops. Median DPLs of CT and MT troops during the study period. The upper and lower quartiles are shown by the range of the ‘box’, median value by the horizontal line within the box, and the full extent of the data given by the ‘whiskers’.

**Figure 5:**
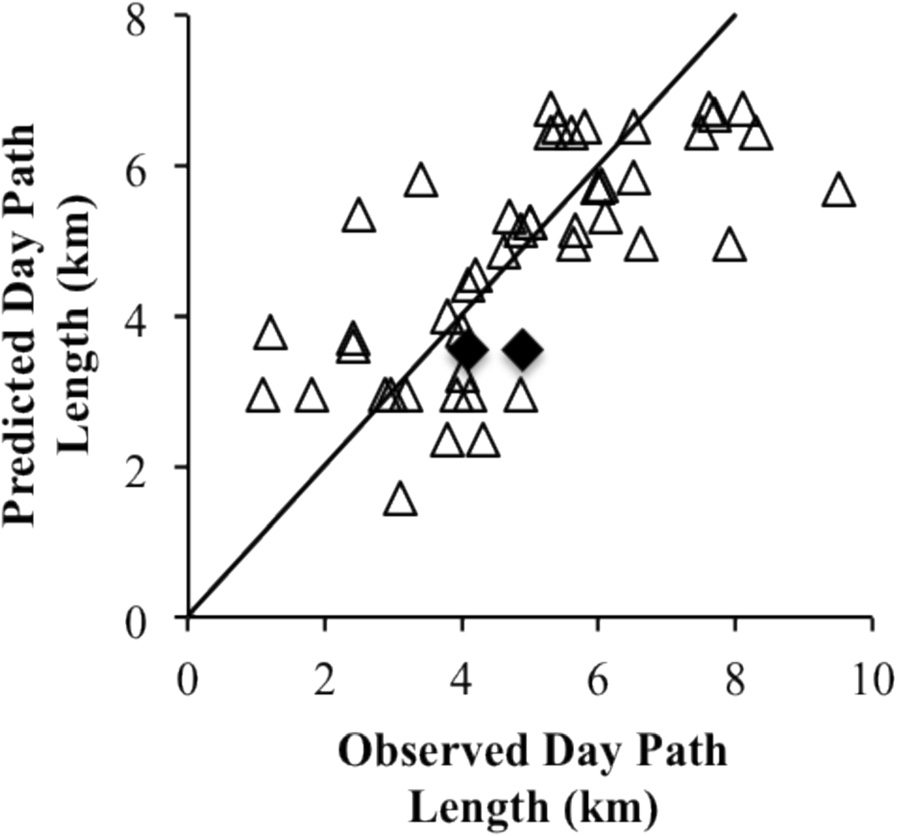
Predicted DPL against observed DPL for baboons on a continental scale. Predicted DPL calculated from a best fitting model model considering the effects of average monthly rainfall, average annual temperature, and anthropogenic influence, for troops listed in Additional file 1. The straight line passing through (0,0) is a hypothetical perfect 1:1 fit between the model and data. Predictions from the model are for N = 47 troops with data for the Issa troops (Ugalla, current study) omitted; observed DPLs for the Issa troops are shown by filled diamonds.

At a local scale, we found that the median DPL for CT and MT were 4.7 km (range: 3.1–8.5) and 4.3 km (range: 1.5–6.0) respectively (Figure 4), and there was not a statistical difference between the DPLs of the two troops (Mann Whitney *U*-test: n_CT_ = 22, n_MT_ = 12, P = 0.725). Comparison of these observed DPLs and those DPLs predicted by the best continental-level model (see above) that considers monthly rainfall, annual temperature, and anthropogenic influence, whilst accounting for population, revealed that the actual DPL of Issa baboons was similar to the predicted DPL (Figure 5). Therefore, it appears that yellow baboons at Issa are not atypical and the same ecological factors that impact on baboon troop DPLs throughout their range are also good predictors of Issa troops DPLs.

**Figure 6:**
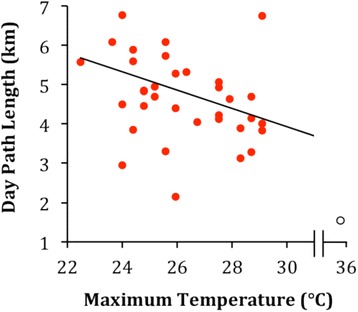
Relationship between maximum daily temperature and DPL in Issa baboons. The fitted line represents DPL as predicted by temperature (LMM: effect[SE] = −235.8[104.8]; df = 1; p = 0.025; see Table 4 for full model results). The empty circle to the right hand side represents the highest daily temperature and lowest DPL reading recorded; removing this data point does not quantitatively change the model results.

Consideration of local ecological factors revealed that Issa baboon troops travelled significantly further (Table 4; Figure 6 and see Additional file 3 for best candidate models) and faster (Table 5) on cooler days. Due to the sensitivity of the vertebrate brain to even slight changes in temperature, the need for primates to regulate their internal temperature is vital [96]. In order to cool the brain, baboons dissipate heat through panting [97], however, they lack more typical mechanisms for the effective cooling of the brain (i.e. carotid rete) that are present in other similar sized, sympatric mammals [98]. This likely makes high radiant temperatures a greater challenge to their thermoregulation [98]. To avoid overheating, baboons have been observed to adjust their activity according to their thermoregulatory needs, with temperature being a negative function of activity in hot environments [33,61,87,99]. During periods of intense thermal loading, baboons are found to respond by seeking shade and engaging in more sedentary behaviours such as resting and grooming [32,87,100]. Similarly, Stelzner [99] found that travel rate in Amboseli baboons was dependent on ambient temperature at a microhabitat type level, and on hot days the baboons would slow down when traversing more shaded areas. It is plausible then, that as heat stress increases, baboons at Issa are forced into more sedentary activities, which could result in the reduced DPLs and speeds we observed. Concurrent direct observations of individual and troop level behaviours would be required to confirm that Issa baboons move less on hotter days due to enforced resting.

**Table 4:** **Estimate, standard error, test statistic and P-value for predictors of baboon troop DPL at a local scale**

**Model term**	**Estimate**	**Standard error**	***t-*** **value**	***df***	***p***
Max. temperature	**−261.8**	**75.2**	**−3.48**	**1**	**0.0017**
Fruit Abundance Index	27.6	204.7	0.14	1	0.89
Season (dry, wet)^1^	−512.61	408.24	−1.26	1	0.22
Troop ID (CT, MT)^2^	−276.18	405.24	−0.68	1	0.50

The best fitting model included those terms shown in bold text; for AIC values of the best candidate models tested see Additional file 3.

^1^Reference category was wet season. ^2^CT = Camp Troop. MT = Matawi Troop; reference category was Camp Troop.

**Table 5:** **Estimate, standard error, test statistic and P-value for predictors of baboon troop travel speed at a local scale**

**Model term**	**Estimate**	**Standard error**	***t*** **-value**	***df***	***p***
Max. temperature	**−0.04**	**0.01**	**−2.79**	**1**	**0.005**
Fruit Abundance Index	**−**0.003	0.03	**−**0.09	1	0.93
Season (dry, wet)^1^	0.004	0.14	0.027	1	0.98
Rainfall	0.007	0.005	1.43	1	0.16
***Smoothing factor***		***F***	**edf**	**rdf**	***p***
Day		0.16	1	1	0.69

The best fitting model included those terms shown in bold text. Effect of smoothing factor is also shown with estimated degrees of freedom (edf), reference degrees of freedom (rdf), test statistic (*F*) and *p* value.

^1^Reference category was wet season.

Contrary to our expectations, we did not find FAI to significantly affect either DPL (Table 4 see Additional file 3 for best candidate models) or the movement characteristics of baboons at Issa (Table 5; Table 6). A critical influence on ranging patterns of *P. cynocephalus* is the distribution of foods [9]. In contrast with other studies [9,10] local fruit abundance (here, FAI) did not significantly predict DPL (Table 4). Our finer resolution analysis of the baboon’s movement characteristic similarly found no effect of FAI on speed or turning angles. This is surprising, as primates have been consistently shown to use the space in their habitats according to the learned locations of particular resources and consequently move efficiently between them [48-51]. This is especially true of fruiting trees, a core food group for baboons [12,33,37-40,76-80]. In support of this, Noser & Byrne [101] found baboons demonstrated increased route linearity and speed when travelling to sparse, out of site, fruit patches indicating the tendency for baboons to use their space in an efficient, goal-directed way. For this reason, we expected Issa baboons to demonstrate more direct travel movements when fruit availability increases. The difference between the two studies is instructive, and highlights the need for combining behavioural (or at least basic activity data) with movement information, so that it is possible to analyse segments of travel between known resources [101]. We therefore proceeded to explore whether FAI and/or season predicted the time troops spent moving (i.e. speeds of <1 m/min versus >1 m/min). We reasoned that time spent feeding should decrease with proportion of carbohydrate-rich fruits [101,102] in the diet [30] resulting in decreased moving time as compared to other time budget variables [102]. Therefore, we expected to see less time spent moving during periods of high FAI, and our model (Additional file 4) confirmed this to be the case. Thus, whilst fine-scale movement of Issa baboons was not predicted by the availability of fruit resources, fruit availability did fundamentally alter the time they spent moving [4,5,17].

**Table 6:** **Estimate, standard error, test statistic and P-value for predictors of baboon troop turning angle at a local scale**

**Model term**	**Estimate**	**Standard error**	***t*** **-value**	***df***	***p***
Max. temperature	−0.008	0.009	−0.98	1	0.33
Fruit Abundance Index	−0.0006	0.03	−0.19	1	0.98
Season (dry, wet)^1^	−0.14	0.072	−1.93	1	0.055
Rainfall	−0.00009	0.003	−0.04	1	0.97
***Smoothing factor***		***F***	**edf**	**rdf**	***p***
Day		1.98	1	1	0.16

Effect of smoothing factor is also shown with estimated degrees of freedom (edf), reference degrees of freedom (rdf), test statistic (*F*) and *p* value.

^1^Reference category was dry season.

We found no significant effect of season (wet, dry) on baboon DPLs or movement characteristics (Tables 4, 5 and 6), although the effect of season on the distribution of turning angles was P = 0.055 (Table 6), indicating a trend for troops’ movements to become more direct during the dry season in line with our original predictions. It may be possible that the lack of any strong seasonal patterns on movement characteristics may be due to the availability of water. Baboons are obligate drinkers [9] relying heavily on surface water, the availability of which is subject to large variation in sub-Saharan Africa. Surface water is therefore an important determinant of baboon ranging patterns [37], and its availability is ultimately determined by seasonal rainfall [40] (also see above continental model). During our study period, surface water was readily available to the baboons, and so was unlikely to constrain movement paths. However, our study period did not extend through the driest months at the end of the dry season when running water at Issa becomes stagnant and gradually more confined to water holes [103]. Thus, the influence of surface water availability on ranging patterns cannot be fully determined without further study.

There may well be other key ecological factors that are important drivers of Issa baboon movements that we did not measure. For example, baboons mitigate the serious threat of nocturnal predation by utilising sleeping sites (i.e. specific sleeping trees or cliffs) [17,104], and it is possible that the lower limit of DPL is set by the troops having to reach or travel between these sleeping sites [13,105,106]. Also relevant is the capacity of predation, especially by ambush predators, to influence ranging behaviour of primates [107]. Areas perceived to be ‘high-risk’ (vegetation allowing predators to conceal their approach) are commonly avoided by baboons [108], and leopards (*Panthera pardus*), the primary predator of baboons [109], were encountered frequently at Issa [110]. Their impact on the movement ecology of Issa baboons may be significant [107], and this offers yet another interesting area for future research.

## Conclusions

Overall, this study emphasises the ability of baboons to adapt their ranging behaviour to extrinsic variables [111], and provides much needed data on baboon space-use from a woodland context. This adaptability is reflected, at least in part, by the ubiquity of baboons across a multitude of ecological and climatological contexts throughout sub-Saharan Africa (e.g. from the forests of Gombe in Tanzania, to the deserts of Tsaobis in Namibia). At a continental scale, we demonstrate the importance of including the role of human derived food sources in predicting the ranging patterns of baboons [22]. Human-derived foods are becoming increasingly available to baboons as the distinction between “wild” and “human” landscapes becomes blurred [112], and this factor, it seems, has a stronger effect upon variance in DPLs than group size, for example [29]. Moreover, this study highlights how investigations of movement patterns at different spatial and temporal scales can provide a fuller analysis of the ecological determinants of movement. Site-specific considerations in particular are important, for example, temperature. At a continental scale, baboons in hotter places travel further, whilst baboons on a local scale travel less far on hotter days. In this instance, we find the role of temperature changes depending on the spatial scale at which it is investigated.

## Additional files

Additional file 1:
**Ecological data for DPL continental comparison model.**
Additional file 2:
**Akaike Information Criteria (AIC values for the top ten candidate models that predict variation in annual mean DPL (continental scale).**
Additional file 3:
**Akaike Information Criteria (AIC) values for the top ten candidate models that predict variation in DPL (local scale).**
Additional file 4:
**Model testing variables predicting travel speed of baboon troops (local scale).**

## Abbreviations

CT: Camp Troop
MT: Matawi Troop
DPL: Day path length
GAM: Generalized additive model
LMM: Linear mixed model
AIC: Akaike’s information criterion
PPI: Primary productivity index
GPS: Geographical positioning system
Pann: Average annual rainfall
Tann: Average annual temperature
PmoSD: Standard deviation across average monthly values for 12 months
TmoSD: Standard deviation across average monthly values for 12 months
P < 100: Number of months with less than 100 mm of rainfall
