# Supplementary material for: The ecological determinants of baboon troop movements at local and continental scales
Source: Mov Ecol. 2015 Jul 1;3(1):14. doi: 10.1186/s40462-015-0040-y (PMC4487562; doi:10.1186/s40462-015-0040-y)
Supplement: Additional file 1: — Ecological data for DPL continental comparison model. [file 40462_2015_40_MOESM1_ESM.docx]

**Additional file 1**. Ecological data for each of the n=47 troops used in the DPL continental comparison model.

| **Study Site & (Troop)** | **Troop size** | **Mean Annual DPL** | **GPS Sample Interval (mins)** | **DPL Sample Size** | **Reference** |
| --- | --- | --- | --- | --- | --- |
| Amboseli, Kenya (1) | 59 | 6.10 | 60 | >12months | [71] |
| Amboseli, Kenya (2) | 40 | 4.20 | 60 | >12months | [17] |
| Amboseli, Kenya (3) | 63 | 4.66 | 60 | >12months | [33] |
| Amboseli, Kenya (4) | 55 | 5.43 | 60 | >12months | [33] |
| Amboseli, Kenya (5) | 55 | 4.00 | 60 | >12months | [33] |
| Amboseli, Kenya (6) | 53 | 5.00 | 60 | >12months | [46] |
| Awash, Ethiopia (1) | 160 | 7.50 | NA | >12months | [43] |
| Awash, Ethiopia (2) | 210 | 8.30 | 15 | >12months | [45] |
| Awash, Ethiopia (3) | 81 | 5.30 | 30 | <12months | [74] |
| Awash, Ethiopia (4) | 51 | 6.50 | 30 | <12months | [74] |
| Awash, Ethiopia (5) | 57 | 5.40 | 30 | <12months | [74] |
| Blouberg, SA | 25 | 7.70 | 5 | >12months | (Noser, pers comm) |
| Bole Valley, Ethiopia | 19 | 1.20 | 15 | <12months | [65] |
| Budongo, Uganda | 38 | 3.80 | NA | Unknown | [66] |
| Cape Peninsula, South Africa (1) | 49 | 5.64 | 20 | >12months | [125] |
| Cape Peninsula, South Africa (2) | 36 | 4.85 | 20 | >12months | [125] |
| Cape Peninsula, South Africa (3) | 22 | 6.61 | 20 | >12months | [125] |
| Cape Peninsula, South Africa (4) | 16 | 1.80 | 20 | >12months | [125] |
| Cape Peninsula, South Africa (5) | 26 | 3.17 | 20 | >12months | [125] |
| Cape Peninsula, South Africa (6) | 16 | 3.92 | 20 | >12months | [125] |
| Cape Peninsula, South Africa (7) | 35 | 4.13 | 20 | >12months | [125] |
| Cape Peninsula, South Africa (8) | 24 | 2.89 | 20 | >12months | [125] |
| Cape Peninsula, South Africa (9) | 115 | 2.96 | 20 | >12months | [125] |
| Cape Peninsula, South Africa (10) | 85 | 7.90 | 30 | >12months | [77] |
| Chololo, Kenya | 102 | 5.60 | 15 | Unknown | [67] |
| Drakensberg, SA (1) | 9 | 4.30 | 30 | <12months | [78] |
| Drakensberg, SA (2) | 14 | 3.80 | 30 | >12months | [78] |
| Erer-Gota, Ethiopia | 72 | 9.50 | NA | >12months | [75] |
| Gashaka Gumti, Nigeria (1) | 19 | 3.10 | 30 | >12months | [68] |
| Gashaka Gumti, Nigeria (2) | 28 | 2.40 | 30 | >12months | [68] |
| Gilgil, Kenya | 57 | 4.60 | NA | <12months | [12] |
| Honnet, SA (1) | 77 | 4.00 | NA | >12months | [79] |
| Honnet, SA (2) | 59 | 6.50 | NA | >12months | [79] |
| Ishasha, Uganda | 45 | 2.40 | 60 | >12months | [69] |
| Metahara, Ethiopia | 87 | 5.80 | NA | <12months | [70] |
| Mikumi, Tanzania | 120 | 3.40 | NA | >12months | [72] |
| Mkuzi, SA | 71 | 6.04 | 10 | Unknown | [42] |
| Mt. Assirik, Senegal (1) | 250 | 8.10 | 30 | >12months | [38] |
| Mt. Assirik, Senegal (2) | 135 | 7.60 | 30 | >12months | [38] |
| Mulu, Ethiopia | 22 | 1.10 | NA | Unknown | (Dunbar, pers comm) |
| Suikersbosrand, South Africa | 78 | 4.10 | NA | >12months | [81] |
| Tana, Kenya | 75 | 5.30 | 15 |  | [73] |
| Tsaobis, Namibia (1) | 57 | 6.00 | 30 | >12months | [44] |
| Tsaobis, Namibia (2) | 32 | 6.00 | 30 | >12months | [44] |
| DeHoop, South Africa (1) | 40 | 5.65 | 30 | <12months | [80] |
| DeHoop, South Africa (2) | 17 | 4.86 | 30 | <12months | [80] |
| Mt. Zebra, South Africa | 28 | 2.50 | NA | Unknown | [82] |
| Issa, Tanzania | 24 | 4.70 | 5 | <12months | Current study |
| Issa, Tanzania | 32 | 3.70 | 5 | <12months | Current study |
