# Supplementary material for: The ecological determinants of baboon troop movements at local and continental scales
Source: Mov Ecol. 2015 Jul 1;3(1):14. doi: 10.1186/s40462-015-0040-y (PMC4487562; doi:10.1186/s40462-015-0040-y)
Supplement: Additional file 2: — Akaike Information Criteria (AIC values for the top ten candidate models that predict variation in annual mean DPL (continental scale). [file 40462_2015_40_MOESM2_ESM.docx]

**Additional file 2**

Akaike Information Criteria (AIC) values for the top ten candidate models that predict variation in annual mean DPL at a continental scale. Candidate models are based on compatible effects, in ascending order of AIC value. The model in bold indicates the final model selected.

| Candidate models | df | AIC |
| --- | --- | --- |
| Tann + Pann + Ant + Samp | 8 | 168.6 |
| Tann + P<100 + Samp + Ant | 7 | 169.0 |
| Tann + P<100 + Samp + Ant + GS | 8 | 169.1 |
| Tann + Pann + Ant + Samp + Alt | 9 | 169.2 |
| Tann + Pann + Ant | **6** | **169.5** |
| GS + Pann + Ant + Samp + Alt | 8 | 169.9 |
| GS + PPI + Ant + Samp + Alt + Pr | 9 | 170.0 |
| Ant + Samp + NDVI + P<100 + Tann | 8 | 170.6 |
| Ant + Samp + Alt + P<100 + Tann | 8 | 170.6 |
| Ant + Samp + NDVI + PmoSD + Tann + TmoSD | 9 | 171.4 |

*Alt* altitude (meters above sea level), *Pr* primate species count at study site, *Ant* troops exposed to anthropogenic influence, *Pann* average annual rainfall, *PmoSD* standard deviation for average monthly rainfall (mm), *Tann* average annual temperature (°C), *TmoSD* standard deviation for average monthly temperature (°C), *PPI* primary productivity index (number of months in the year in which rainfall was twice the average annual temperature), *P<100* number of months with less than 100mm rainfall, *NDVI* normalised difference vegetation index retrieved from remote sensing data, *Samp* sample size (>12 months, <12 months or unknown).
