# Supplementary material for: The ecological determinants of baboon troop movements at local and continental scales
Source: Mov Ecol. 2015 Jul 1;3(1):14. doi: 10.1186/s40462-015-0040-y (PMC4487562; doi:10.1186/s40462-015-0040-y)
Supplement: Additional file 3: — Akaike Information Criteria (AIC) values for the top ten candidate models that predict variation in DPL (local scale). [file 40462_2015_40_MOESM3_ESM.docx]

**Additional file 3**

Akaike Information Criteria (AIC) values for the top ten candidate models that predict variation in DPL at a local scale. Candidate models are based on compatible effects, in ascending order of AIC value. The model in bold indicates the final model selected.

| Candidate models | df | AIC |
| --- | --- | --- |
| **Max Temp** | **1** | **418.4** |
| Max Temp + Se | 2 | 418.7 |
| Max Temp + FAI | 2 | 419.2 |
| Max Temp + Tr | 2 | 419.9 |
| Max Temp + Tr + Se | 3 | 420.1 |
| Max Temp + Tr + FAI | 3 | 420.9 |
| Max Temp + Se + Tr + FAI | 4 | 422.1 |
| Tr + Se | 2 | 430.6 |
| Se + FAI | 2 | 431.1 |
| Tr + Se + FAI | 3 | 432.6 |

*FAI* fruit availability index, *Max Temp* maximum temperature, *Se* season, *FAI* fruit abundance index, *Tr* troop ID
