# Supplementary material for: The ecological determinants of baboon troop movements at local and continental scales
Source: Mov Ecol. 2015 Jul 1;3(1):14. doi: 10.1186/s40462-015-0040-y (PMC4487562; doi:10.1186/s40462-015-0040-y)
Supplement: Additional file 4: — Model testing variables predicting travel speed of baboon troops (local scale). [file 40462_2015_40_MOESM4_ESM.docx]

**Additional file 4**

To test what factors predicted activity in baboons (i.e. not moving, or moving) we fitted a GAM with a binomial error structure (mgcv package, R: [67]). Our response was defined by speed with <1m/min defined as not moving, and moving defined as >1m/min. Since we observed temporal auto-correlation in our data, we randomly sub-sampled n=30 data points from each observation day to remove this effect. We then fitted FAI, season (wet, dry), rainfall and maximum temperature as fixed effects, whilst controlling for any effect of day (of study period). We then selected the best model according to the lowest AIC value [69], but models within two AIC points were considered to be plausible alternatives and the model that was the most parsimonious (i.e. the model with the fewest fixed effects) was selected preferentially [70]. The results of our analyses are provided in Table below.

Estimates, standard error, test statistics and P-value for predictors of activity (not moving, or moving; <1m/min versus >1 m/min) in the selected General Additive Model at a local scale. The best fitting model included those terms shown in bold text. Effect of smoothing factor is also shown with estimated degrees of freedom (edf), reference df, chi squared value (Chi.sq) and *p* value.

| Model term | | Estimate | | Standard error | | *z*-value | *df* | *p* |
| --- | --- | --- | --- | --- | --- | --- | --- | --- |
| Fruit Abundance Index | | -0.51 | | 0.13 | | -3.85 | 1 | <0.001 |
| Max. temperature | | -0.008 | | 0.02 | | -0.31 | 1 | 0.76 |
| Season (dry, wet)^1^ | | 0.45 | | 0.42 | | 1.11 | 1 | 0.29 |
| Rainfall | | 0.0004 | | 0.007 | | 0.05 | 1 | 0.05 |
| *Smoothing factor* | | | **edf** | **Ref.df** | **Chi.sq** | | ***p*** | |
| Day | 7.69 | | | 8.58 | 56.53 | | <0.0001 | |

^1^ Reference category was wet season.
